# The mediating role of resilience in the relationship between stress and life satisfaction among Chinese medical students: a cross-sectional study

**DOI:** 10.1186/s12909-015-0297-2

**Published:** 2015-02-13

**Authors:** Meng Shi, XiaoXi Wang, YuGe Bian, Lie Wang

**Affiliations:** 1 English Department, School of Basic Medicine, China Medical University, 92 North 2nd Road, Heping District, Shenyang, Liaoning PR China; 2 School of Applied Technology, China Medical University, 92 North 2nd Road, Heping District, Shenyang, Liaoning PR China; 3 Drury University graduated student, 92 North 2nd Road, Heping District, Shenyang, Liaoning PR China; 4 Department of Social Medicine, School of Public Health, China Medical University, 92 North 2nd Road, Heping District, Shenyang, Liaoning PR China

**Keywords:** Stress, Life satisfaction, Resilience, Medical students

## Abstract

**Background:**

The psychological distress of medical students has been widely acknowledged. However, few studies focused on positive well-being among medical students. The purpose of this study was to investigate related demographic factors of life satisfaction among Chinese medical students, to examine the relationship between stress and life satisfaction among this group of people, and to explore the mediating role of resilience in this relationship.

**Methods:**

This multicenter cross-sectional study was carried out in June 2014. Self-reported questionnaires consisting of Perceived Stress Scale (PSS), Wagnild and Young Resilience Scale (RS-14), Satisfaction with Life Scale (SWLS), as well as demographic section were distributed to students at four medical colleges and universities in Liaoning province, China. A total of 2925 students (effective response rate: 83.6%) became our subjects. Hierarchical linear regression models were used to explore the mediating role of resilience.

**Results:**

Among the demographic factors, life satisfaction was significantly different in gender (P = 0.001) and study programs (P < 0.001). Stress was negatively correlated with life satisfaction (r = −0.35, P < 0.01). After adjusting for the demographic factors, stress accounted for 12% of the variance in life satisfaction (β = −0.34, P < 0.001) while resilience explained an additional 18% of the variance (β = 0.46, P < 0.001). Resilience functioned as a partial mediator in the relationship between stress and life satisfaction among Chinese medical students.

**Conclusions:**

Both stress and resilience played a big role in life satisfaction among Chinese medical students. Besides reducing perceived stress, the university authorities should adopt evidence-based intervention strategies to enhance their resilience in order to promote life satisfaction among the students.

## Background

Medical education is stressful for many students [1,2]. While previous literatures mainly focused on mental and psychological distress among medical students, such as depression and anxiety [3,4], few studies explored their positive well-being. Even though many studies have examined positive well-being among different groups of people [5-8], especially among patients with chronic illnesses [9-11], the research of this issue among medical students is underrepresented. It has been widely recognized that psychological distress among medical students could lead to negative health and social consequences, such as poor academic performance, increased rates of substance use, and suicide [12-15]. Besides, it is also related to quality of health care they will provide in the future [16]. Thus, it is not uncommon that many relevant studies proposed intervention strategies on the prevention and treatment of these conditions.

However, it is of equal importance to promote the positive well-being among medical students. In a meta-analysis by Howell et al. [17], ill-being was found to slightly more strongly predict short-term health outcomes, while positive well-being slightly more strongly predicted long-term health outcomes, but the effects were of similar size. Positive well-being is not simply the absence of negative well-being, as the two constructs involve distinct activation of physiological and neurobiological systems [18]. Positive well-being is composed of life satisfaction, positive affect and negative affect [19]. Life satisfaction is a major component of positive well-being and may be more stable than measures of affects [20]. It has been shown to have protective effects on all-cause mortality in prospective cohort studies [21,22]. In a large sample of young adults in 21 nations, life satisfaction was also found to be related to people’s positive behaviors, such as physical exercise, not smoking, healthier diet [23]. In addition, another large-scale study revealed that life satisfaction was negatively associated with suicide rate in a sample of 32 nations [24].

The negative consequences of high stress in medical education are well recognized, such as decreased attention, depression and anxiety, poor relationships, and even suicide [25]. Although stress has often been considered risk factor of psychological distress among medical students, many students exposed to the same level of stress do not develop psychopathology and these students are believed to possess resilience. Resilience refers to the capacity and dynamic process of adaptively overcoming stress and adversity while maintaining normal psychological and physical functioning [26]. Prior research has revealed that resilience was negatively related to stress [27,28], and resilient individuals used positive emotions to alleviate the effects of stress and showed physiological differences in their capability to adapt to stress [29,30]. When faced with a stressor, they not only rebound quickly, but also grow stronger in the process [31]. On the other hand, resilience was shown to be positively correlated with life satisfaction [32,33]. Resilient physicians were found to better control their working hours and schedules, which was the strongest predictor of life-work balance [34]. Based on transactional stress theory [35], personality characteristics, such as resilience, influence appraisal process which in turn mediates the stressor-stress response relationship. However, to our knowledge, no study has yet been conducted to explore the mediating role of resilience in the relationship between stress and life satisfaction.

Medical students play vital roles in health care service in the future. While the society of China witnessed dramatic urbanization process and enrollment expansion of college students in the past decade, no study on their life satisfaction has been performed. Therefore, we carried out this multi-center study with the following aims: 1) to investigate the related demographic factors of life satisfaction among Chinese medical students; 2) to examine the relationship between stress and life satisfaction; 3) to explore the mediating role of resilience in the relationship between stress and life satisfaction.

## Methods

### Research design and sample

This cross-sectional study was conducted in Liaoning province (with a population of roughly 44 million), in northeast region of China in June 2014. All the four medical tertiary institutions in the province were included in the investigation, which were China Medical University, Dalian Medical University, Liaoning Medical College and Shenyang Medical College respectively. The medical education in China mainly consists of 5-year programs, 7-year programs and postgraduate education. In the current study, we focused on clinical medicine students in 5-year programs and 7-year programs. For the students in 5-year programs, they are awarded bachelor degrees in medicine upon graduation, whereas those in 7-year programs are awarded master degrees in medicine upon graduation. As more medical students enrolled in 5-year programs, 4 whole classes of clinical medicine students in 5-year programs and 3 whole classes of clinical medicine students in 7-year programs were randomly chosen from each institution based on each academic year. The number of students in each class in Chinese medical school usually ranges from 25 to 40 in both programs. Self-administered questionnaires were either distributed to the students in the last 20 minutes or so in class time or sent to the students of higher grades who were on the stage of clinical practice in teaching hospitals by site coordinators. A total number of 3500 questionnaires were distributed and 3095 (88.4%) students returned the questionnaires in class or before the deadline. 170 invalid questionnaires (≥20% items unanswered) were excluded and a pool of 2925 students (effective response rate: 83.6%) became our final subjects.

Participation in the study was voluntary and the involved students signed informed written consent before their participation. This study was approved by the Committee on Human Experimentation of each institution before the participation of their students and the study procedures were in accordance with ethical standards.

### Measurement of stress

The 10-item Perceived Stress Scale (PSS), developed by Cohen et al. [36], was used to measure the perception of stress among the students. The scale consists of six negative and four positive items that include questions about one’s feelings and thoughts in the previous month, such as “in the last month, how often have you felt confident about your ability to handle your personal problems?” and “in the last month, how often have you found that you could not cope with all the things that you had to do?”. Each item is scored on a 5-point Likert scale, ranging from 0 (never) to 5 (very often). The overall score ranges from 0 to 40 and higher score indicates higher level of perceived stress. The scale has been widely used among Chinese people and demonstrated adequate reliability and validity [37-40]. In current study, the Cronbach’s alpha value for this scale was 0.73.

### Measurement of resilience

Resilience was assessed with the 14-item Wagnild and Young Resilience Scale (RS-14), which is one of the most reliable tools to measure resilience [41]. It is a 7-point Likert scale used in various age groups and on different conditions [42-44]. The examples of the items are “I am determined” and “when I’m in a difficult situation, I can usually find my way out of it”. Each item is graded from 1(strongly disagree) to 7 (strongly agree). Graded items are summed up to provide a total score and lower scores indicate less resilience. The Cronbach’s alpha of the scale was 0.94 in this study.

### Measurement of life satisfaction

To measure global life satisfaction, we used Satisfaction with Life Scale (SWLS), which was developed by Diener et al. [45]. The scale consists of five items, which include “in most ways my life is close to my ideal” and “the conditions of my life are excellent”. The respondents are asked to answer each item on a 7-point Likert scale from 1 (strongly disagree) to 7 (strongly agree). Answers are added to create overall score from 5 to35, with higher score indicating higher level of life satisfaction. Previous research demonstrated satisfactory content and predictive validity of the scale among various age groups [45-47]. In the present study, the Cronbach’s alpha value for this scale was 0.92.

### Demographic characteristics

Demographic information regarding age, gender, place of residence, study programs, academic year, and educational levels of both parents was obtained in this study. Place of residence was dichotomized into rural and urban areas. Study programs were divided into 5-year program and 7-year programs. Educational levels of parents were categorized into primary school, secondary school, college and above.

### Statistical analysis

All analyses were performed using SPSS statistical software for Windows version 17.0 (SPSS, Inc., Chicago, IL). All statistical tests were two-sided and the significance level was set at p < 0.05. Descriptive statistics of the demographic and other variables were indicated with mean, standard deviation (SD), number (N) and percentage (%) as appropriate. T-tests and one-way ANOVA were used to compare differences in life satisfaction among categorical groups. Pearson’s correlation was used to examine correlations among continuous variables. Hierarchical regression analysis was used to explore the mediating effects of resilience in the relationship between stress and life satisfaction. Based on Baron and Kenny’s technique on mediation [48], the following conditions should be met. (1) the independent variable (stress) is significantly related to the dependent variable (life satisfaction); (2) the independent variable (stress) is significantly related to the mediator (resilience); (3) the mediator (resilience) is significantly related to the dependent variable (life satisfaction), with the effect of the independent variable (stress) on the dependent variable (life satisfaction) shrinking (partial mediator) or becoming statistically insignificant (full mediator) upon the addition of the mediator (resilience) to the model. Standardized estimate (β), F, R^2^, and R^2^-changes (△R^2^) for each step were provided. Tolerance and variance inflation factor were used to check for multicollinearity. Moreover, Sobel test was performed to calculate the mediation effect.

## Results

### Characteristics of subjects

The basic characteristics of the medical students and the distributions of life satisfaction in categorical variables are shown in Table 1. Among the 2925 students, 1028 (35.1%) were males, while 1897 (64.9%) were females. Their age ranged from 15 to 28 (M =21.7, SD = 1.95). Compared with male students, female students had a significantly higher level of life satisfaction (P < 0.05). Compared with 5-year programs, the students studying in 7-year programs showed a higher score of life satisfaction (P < 0.05). Meanwhile, the differences of life satisfaction in place of residence, paternal and maternal education were not statistically significant (P > 0.05).

**Table 1:** **Demographic characteristics and differences in life satisfaction (N = 2925)**

Variables	N	%	SWLS (Mean ± SD)	P
Gender				
Male	1028	35.1%	23.5 ± 6.86	0.001
Female	1897	64.9%	24.3 ± 6.77	
Age				
15-21	1406	48.1%	24.2 ± 6.80	0.137
22-28	1519	51.9%	23.9 ± 6.83	
Place of Residence				
Urban area	1809	61.9%	24.2 ± 6.87	0.165
Non-urban area	1116	38.1%	23.8 ± 6.72	
Paternal education				
Primary school	301	10.3%	23.6 ± 6.78	0.413
Middle School	1450	49.6%	24.0 ± 6.63	
College and above	1174	40.1%	24.2 ± 7.05	
Maternal Education				
Primary school	469	16.0%	23.8 ± 6.59	0.183
Middle School	1524	52.1%	23.9 ± 6.78	
College and above	932	31.9%	24.4 ± 6.98	
Study program				
Five years	1738	59.4%	23.3 ± 7.00	<0.001
Seven years	1187	40.6%	25.1 ± 6.39	

SWLS: Satisfaction with Life Scale.

### Correlations among study variables

The mean, standard deviations of continuous variables and Pearson correlation analyses are presented in Table 2. Results revealed that all psychological variables were significantly correlated with each other, and those correlations were in the expected direction. Age was only significantly correlated with resilience (r = −0.08, P < 0.01). Specifically, stress was negatively correlated with resilience (r = −0.36, P < 0.01) and life satisfaction (r = −0.35, P < 0.01). Resilience was positively correlated with life satisfaction (r = 0.53, P < 0.01). Therefore, the first two conditions of Baron and Kenny’s technique were satisfied in the present study.

**Table 2:** **Means, standard deviation (SD) and correlations of continuous variables**

Variables	Mean	SD	1	2	3	4
1. Age	21.7	1.95	1			
2. Stress	17.1	5.10	0.02	1		
3. Resilience	69.3	15.66	−0.08^**^	−0.36^**^	1	
4. Life satisfaction	24.0	6.82	−0.01	−0.35^**^	0.53^**^	1

**P < 0.01 (two-tailed).

### The mediating role of resilience in the relationship between stress and life satisfaction

The hierarchical linear regression analyses for exploring the mediating role of resilience are demonstrated in Table 3. After adjustment for gender and study programs in step 1, independent variable of stress was added in step 2; then resilience was entered into step 3 of the model. The results revealed that stress was negatively associated with life satisfaction, explaining 12% of the variance (β = −0.34, P < 0.001), while resilience was positively associated with life satisfaction, accounting for an additional 18% of the variance (β =0.46, P < 0.001). In addition, resilience played a partial mediating role in the association between stress and life satisfaction as absolute value of its standardized regression coefficient (β) reduced from 0.34 to 0.18 (Sobel test, z = −16.77, P < 0.001). Tolerance (range: 0.85–0.99) and variance inflation factor (range: 1.01–1.17) did not indicate a multicollinearity problem.

**Table 3:** **Hierarchical linear regression analyses results**

Variables	Step 1 (β)	Step2 (β)	Step3 (β)
Gender	0.07**	0.06**	0.02
Study program	0.14**	0.10**	0.06**
Stress		−0.34**	−0.18**
Resilience			0.46**
F	33.03**	155.03**	335.62**
R^2^	0.02	0.14	0.32
△R^2^	0.02	0.12	0.18

**p < 0.01 (two-tailed).

## Discussion

To our knowledge, this is the first study to investigate the status of life satisfaction among Chinese medical students, and the first one to examine the mediating role of resilience in the relationship between stress and life satisfaction among medical students. The large sample of students from all the four medical tertiary institutions in Liaoning province and the high effective response rate seemed to increase the generalization of our study. Age was found to be negatively associated with resilience among the psychological variables, even though the effect size was rather small. It could be attributed to fact that as the age of the medical students increased, they were more likely to face greater stress from various sources, especially job-hunting in competitive labor market. As revealed in the study, perceived stress was negatively related to their resilience. The results showed that among the demographic factors, life satisfaction was only significantly different in gender and study programs, whereas there was no significant difference in the remaining categorical groups. The average score of life satisfaction of female medical students was significantly higher than that of male ones. This result is not consistent with the previous research [32], in which gender failed to predict life satisfaction in Norwegian medical students. This might be due to the traditional Chinese culture in which males have been considered the pillar of family in almost every aspect of life and they face higher expectations since they are very young. They often face greater pressure than females. Thus, it is reasonable that female medical students in China enjoy higher level of life satisfaction. Compared with 5-year programs, the students in 7-year programs demonstrated a higher score. This might be attributed to the following factors. Firstly, better educational resources and various opportunities are often more accessible to the students in 7-year programs. These students can engage in more educational and social activities, better enriching their life and exploiting their potential. In addition, the prospect of landing satisfactory jobs upon graduation for students in 7-year programs is much brighter, as most hospitals in big cities in China today recruit applicants with at least master degrees. Meanwhile, a large proportion of students in 5-year programs have to prepare for postgraduate school entrance examination years ahead to further their medical study. The huge stress of preparing for the competitive entrance examination might lower their life satisfaction to a large extent.

The significant negative correlation between perceived stress and life satisfaction in our study is correspondent with the previous result found among college students [49]. After adjustment for the demographic factors, stress accounted for 12% of the variance in life satisfaction in our study. Based on the transactional model, stress is regarded as a dynamic interaction between the individual and the environment [50]. The model advocates that individuals not only respond to, but also elicit and shape environmental experiences [35]. Therefore, some sound intervention strategies could be incorporated in medical education to help students to better deal with stress. Prior research revealed that mindfulness training could effectively reduce the stress facing medical students in many nations [51-53]. Besides, effective coping with dependent stressors was found to lower stress, while ineffective coping could contribute to, or intensify subsequent stress in young adults [54]. The classroom setting proved to be a feasible place to teach medical students coping strategies to deal with stress [55].

Stress had not only direct effect on life satisfaction, but also indirect effect on it through resilience. Resilience was found to partially mediate the relationship between stress and life satisfaction. The medical students who scored high on perceived stress had lower resilience, resulting in lower levels of life satisfaction, while those who scored low on perceived stress had higher resilience, contributing to higher levels of life satisfaction. The associations between resilience and positive emotions have been confirmed by prior research [29,30]. These positive emotions protect psychological health by undoing or buffering the negative effects of stress [56]. Moreover, resilient people were found to possess a host of psychological resources, including optimism, tranquility, low neuroticism and high openness. They are more likely to find positive meaning in problems they have to face [29]. These attributes of resilient people contribute to higher levels of life satisfaction. The results implied that besides reducing perceived stress, intervention strategies could also focus on cultivation of resilience. The development of resilience is of great value not only to mitigating maladaptive coping and stress , but also to promoting coping mechanisms [57], resulting in higher levels of life satisfaction. According to the theory of social determinants of health, Khanlou and Wray proposed a whole community approach, emphasizing family, school, community and social factors all could make their contributions to resilience building [58]. Luthara and Brownb pointed out that relationships lied at the “roots” of resilience: the presence of support, love, and security fostered resilience by reinforcing people's innate strengths [59]. Therefore, the university authorities could adopt evidence-based measures to enhance the resilience of medical students so as to enhance their life satisfaction. A previous study has demonstrated that the experimental group of college students in a 4-week resilience education program showed significantly higher resilience scores and more effective coping strategies, compared with control group [60]. Another study revealed that employees at a large medical center increased their resilience over a 12-week period by taking part in a self-directed resiliency training program [61]. In addition, some other research also showed that adolescents significantly enhanced their resilience through various resilience intervention programs [62,63]. However, there is a scarcity of relevant intervention research among medical students, which should be conducted in the future.

Several limitations of the present study have to be acknowledged. Firstly, due to the nature of cross-sectional study, we were unable to draw causal relations among study variables. The findings from the current study should be confirmed by prospective cohort studies in the future. Secondly, all data were obtained through self-reported questionnaires, which could introduce response bias. The participants might have underestimated or overestimated the relationship between the study variables. Thirdly, given our study sample, the generalization of the results should be taken with caution. More research should be conducted in other colleges and cultures as well.

## Conclusions

This study showed that gender and study programs were significantly associated with the levels of life satisfaction among Chinese medical students. After adjustment for these two demographic factors, perceived stress accounted for 12% of the variance in life satisfaction. Resilience played a mediating role in the relationship between perceived stress and life satisfaction. Therefore, the relevant university and medical school authorities could undertake evidence-based measures not only to reduce the perceived stress among medical students, but also to enhance their resilience in order to promote their life satisfaction.
